# Coping in Familien mit psychisch erkrankten Mitgliedern

**DOI:** 10.1007/s00278-021-00492-8

**Published:** 2021-02-09

**Authors:** Olaf Reis, Lukas Steigmiller, Carsten Spitzer, Michael Kölch, Andre Knabe

**Affiliations:** 1 Klinik für Psychiatrie, Neurologie, Psychosomatik und Psychotherapie im Kindes- und Jugendalter, Universitätsmedizin Rostock, Gehlsheimer Str. 20, 18147 Rostock, Deutschland; 2 Klinik für Psychosomatische Medizin und Psychotherapie, Universitätsmedizin Rostock, Rostock, Deutschland; 3 Institut für Soziologie und Demographie, Universität Rostock, Rostock, Deutschland

**Keywords:** COVID-19, Familie, Stress, Erkrankung, psychisch, Soziale Unterstützung, COVID-19, Family, Stress, Mental disorder, Social support

## Abstract

**Hintergrund:**

Belastungen und Bewältigungen während des Lockdowns in Familien mit psychisch erkrankten Mitgliedern wurden bisher noch nicht beschrieben.

**Ziel der Arbeit:**

Erste Erkenntnisse hierzu sollen gewonnen werden, um Fragestellungen für weitere Forschungen abzuleiten.

**Material und Methoden:**

Narrative Interviews an einer anfallenden Stichprobe von gesunden Familien (*n* = 4), Familien mit psychisch erkrankten Kindern (*n* = 12) und Familien mit psychisch erkrankten Eltern (*n* = 3). Die quantifizierende Auswertung der von 2 Rater*innen vergebenen In-vivo-Codes in den Kategorien „Probleme“ und „Bewältigung“ erfolgt mithilfe von numerischen Verhältnissen und Detailanalysen der Codes, die zwischen den Familien unterscheiden.

**Ergebnisse:**

Der Lockdown brachte eine große Zahl von Problemen in allen Familien mit sich. Während bei Familien mit kranken Kindern die Einschränkungen das größte Problem sind, berichten Familien mit kranken Eltern v. a. über einen Mangel an Unterstützung. Familien mit erkrankten Mitgliedern berichten häufiger über riskante Bewältigungsmuster, insbesondere Rumination und Schuldabwehr und seltener über protektive Bewältigung, insbesondere die Mobilisierung sozialer Unterstützung.

**Schlussfolgerung:**

Beide Risikogruppen bedürfen gezielter Interventionen, die sich insbesondere auf adaptives Elternverhalten und Mobilisierungsstrategien richten sollten.

## Abstract (en)

**Background:**

Stress and coping during the lockdown in families with mentally ill members have not yet been described.

**Aim of the study:**

First findings on this issue are to be gained in order to derive questions for further research.

**Material and methods:**

Narrative interviews were carried out on a convenient sample of healthy families (*n* = 4), families with mentally ill children (*n* = 12) and families with mentally ill parents (*n* = 3). The quantifying evaluation of the in vivo codes assigned by two raters for the categories “problems” and “coping” was carried out with the help of numerical ratios and detailed analyses of the codes that differentiate between the families.

**Results:**

The lockdown resulted in a large number of problems in all families. While in families with mentally ill children the restrictions were the biggest problem, families with mentally ill parents reported mainly about a lack of support. Families with sick members more often reported about risky coping patterns, in particular rumination and denial, and less often about protective coping, especially the mobilization of social support.

**Conclusion:**

Both risk groups require targeted interventions that focus in particular on adaptive parenting behavior and mobilization strategies.

Die Problematik und Bewältigung der Coronakrise wurden in Familien mit psychisch erkrankten Mitgliedern bisher kaum beforscht. In der vorgestellten Studie wurde nach beidem gefragt, nach besonderen Problemen und Coping-Strategien.

## Einführung

Bereits zu Beginn der freiheitseinschränkenden Maßnahmen zur Bekämpfung der „coronavirus disease 2019“ (COVID-19) wurde diskutiert, inwieweit sich diese sich auf Familienprozesse auswirken (Cluver et al. 2020). Die Folgen der „disruptiven Veränderungen“ auf das familiäre Wohlbefinden wurden schnell als riskant beschrieben, wobei „kaskadenartige“ Dysregulationen alle Ebenen der ganzen Familie betreffen (Prime et al. 2020). Tatsächlich enthielt eine frühe epidemiologische Studie aus Texas Hinweise auf eine zumindest vorübergehende Zunahme intrafamiliärer Gewalt (Piquero et al. 2020).

Für Deutschland gibt es bisher kaum Quellen zum Thema. Einige Hinweise enthalten die Wochenberichte des Deutschen Instituts für Wirtschaftsforschung (DIW), in denen die Fortführung der Befragung des Corona-Online-Meinungs-Panel-Survey-Spezial (COMPASS) zur allgemeinen Lebenszufriedenheit während des Lockdowns mit Items aus dem Sozio-oekonomischen Panel des Jahres 2018 verglichen werden (Huebener et al. 2020). Neben einer allgemeinen Abnahme der Zufriedenheit gibt es differenzielle Wirkungen der Pandemiemaßnahmen. So sinkt die Zufriedenheit insbesondere bei alleinerziehenden Müttern, bei Eltern von jüngeren Kindern, bei Eltern im Homeschooling und solchen, die obendrein noch im Homeoffice arbeiteten. Die Belastung durch Familienarbeit war insgesamt bei den Frauen höher, wobei auch die Männer mehr für die Familie taten als sonst. Bei ihnen war, womöglich bedingt durch die Einführung der massenhaften Kurzarbeit, der proportionale Anstieg an Familienarbeit höher als bei den Frauen (Zinn et al. 2020). Differenzielle Wirkungen der Maßnahmen auf Familien mit psychischen Erkrankungen wurden hingegen bisher kaum beforscht. Nach einer Studie zu depressiven Müttern (Cameron et al. 2020) stiegen während der Pandemie die Prävalenzen dieser Erkrankung. Mütter von Kindergarten- und Vorschulkindern (Alter 18 Monate bis 8 Jahre) waren stärker betroffen als die Mütter von Kleinstkindern.

Studien, die das Zusammenspiel von psychischen Störungen bei Kindern und den Pandemiebedingungen aufklären, sind den Autoren bisher nicht bekannt. Die Neuheit des Phänomens ließ eine „entdeckende Sozialforschung“ (Kleining 1995) notwendig erscheinen, in der Forschungsfragen oder Hypothesen für weitere Studien generiert würden. Aus diesem Grunde wurde ein qualitativer Ansatz gewählt, mit dem Ziel, Problemlagen während des Lockdowns in Familien mit psychisch erkrankten Mitgliedern, in denen die Kinder beschult werden mussten, aufzuklären. Die vorgestellten Analysen widmen sich den Fragestellungen: Welche Probleme werden von Familien berichtet, die einerseits von psychischen Erkrankungen betroffen waren, andererseits in der Pflicht standen, die Kinder zu Hause zu unterrichten? Unterscheiden sich die Problemlagen, je nachdem, ob die psychischen Probleme bei Eltern oder Kinder oder gar nicht auftreten? Welche Bewältigungsstrategien werden verfolgt?

## Stichprobe und Rekrutierung

Die Pandemie machte sich in Rostock mit einer ersten Schulschließung am Freitag, dem 13.03.2020, bemerkbar, eine Woche vor dem bundesweiten Lockdown. Die Akuität des Problems verlangte nach schnellem Forschungshandeln, womit keine Zeit für Antragsprozeduren oder Personalbereitstellung blieb. Vorhandene kostengünstige Ressourcen für die Forschung mussten genutzt werden, um möglichst viele Familien im Homeschooling möglichst zeitnah zum Lockdown zu untersuchen. In Zusammenarbeit von zwei Universitätskliniken mit dem Institut für Soziologie wurde daher eine Woche nach Inkrafttreten des Lockdowns das Design einer qualitativen Studie entworfen, die unter „Low-to-no-budget“-Bedingungen schnell durchführbar sein sollte. Die Hauptidee bestand darin, Lehr- und Forschungstätigkeit zu verbinden sowie die Student*innen der Soziologie als Mitarbeiter*innen für die Befragungen und Verschriftungen einzubeziehen. Der gemeinsam entworfene Interviewleitfaden wurde ab dem 03.04.2020 in einem forschungspraktischen Seminar am Institut für Soziologie und Demographie der Universität Rostock an einer anfallenden Stichprobe (Bekannte der Studierenden) pilotiert. Diese „Lernrunde“ erbrachte 4 Interviews aus Familien, in denen Kinder zu Hause beschult werden mussten. Im zweiten Durchgang sollten die Studierenden die erworbenen Kenntnisse in Patient*innenfamilien anwenden. Jeweils 7 Familien mit schulpflichtigen Kindern sollten aus der Klinik für Psychiatrie, Neurologie, Psychosomatik und Psychotherapie im Kindes- und Jugendalter und der Klinik für Psychosomatische Medizin und Psychotherapie rekrutiert werden. Das Unbedenklichkeitsvotum für die Studie wurde am 06.05.2020 (Nr. A 2020-0103) von der Ethikkommission der Universitätsmedizin Rostock erteilt, womit die Studie ab Mitte Juni ins Feld gehen konnte. Die Interviews wurden nicht mit den Klinikdaten verknüpft. Eltern und Kinder willigten in den Kliniken lediglich ein, dass ihre Kontaktdaten an das Institut für Soziologie weitergegeben wurden. Auf diese Weise wurde sichergestellt, dass die klinisch ungeschulten Interviewer*innen nur das erfuhren, was die Befragten ihnen zu erzählen bereit waren, nachdem sie ihren „informed consent“ gegeben hatten. Das Interview fokussierte auf die Beschreibung und Bewältigung des Lockdowns und war in 5 Abschnitte geteilt, die nacheinander abgearbeitet wurden: 1. Einstieg/Alltag, Soziodemographie, 2. Auswirkungen von COVID-19 und familiäre Regulation, 3. Familienbild, 4. Rolle von psychischen Störungen, 5. Dynamik sozialer Netzwerke (Beitrag von Knabe et al. in diesem Heft). Für die vorgestellten Resultate aus dem zweiten Teil lautete die Instruktion: „Im Folgenden bitte ich Sie zu erzählen, welche Auswirkungen die Coronakrise in Ihrer Familie hatte. Die Auswirkungen können alle Lebensbereiche betreffen; lassen Sie uns mit der Gesundheit beginnen“. Das freie Erzählen der Teilnehmer*innen wurde auf die folgenden Lebensbereiche gelenkt: Gesundheit, Beruf/Einkommen, Partnerschaft/Freunde, Familie, Freizeit und Lebensgefühl. Die immanenten Nachfragen waren: „Wie haben Sie persönlich die Krise erlebt?“ „Auf welche Weise waren Sie von den Einschränkungen betroffen?“ „Wie sind Sie mit den Veränderungen umgegangen?“ und „Wie ist Ihre Familie mit der Situation umgegangen?“

Das erste Interview einer Patientenfamilie fand am 16.05.2020 statt, das letzte am 14.07.2020. Die Rekrutierung im Erwachsenenbereich verlief weniger erfolgreich, weshalb die dort fehlenden Familien aus der Kinder- und Jugendpsychiatrie ergänzt wurden. Damit stehen Interviews aus insgesamt 19 Familien zur Verfügung, von denen 4 nicht erkennbar von psychischer Erkrankung belastet waren, während die Eltern aus 3 Familien in psychotherapeutischer Behandlung waren. In einer dieser Familien gaben die Eltern an, dass auch mindestens ein Kind wegen psychischer Probleme behandelt wurde. Zwölf Familien wurden über die Kinder- und Jugendpsychiatrie rekrutiert; bei keiner dieser Familien ergaben sich Hinweise auf psychiatrische Probleme bei den Eltern.

## Datengewinnung

Die Interviews fanden überwiegend in den Wohnungen der Befragten statt. Die Familien erhielten für ihre Teilnahme am etwa 2‑stündigen Interview 50 €. Die Verschriftung ergab ein Konvolut von ca. 600 Seiten Interviewtext, der für die Analyse in die Software MAXQDA (2020) eingegeben wurde. Die Codierung wurde von 2 Student*innen der Soziologie vorgenommen, die die Vorlesung „Qualitative Methoden“ besucht hatten und in der Zeit vom 01.09.2020 bis zum 31.10.2020 in mindestens wöchentlichen Teamsitzungen (mit Andre Knabe und Olaf Reis) an der Weiterentwicklung des Codesystems arbeiteten. In den Teamsitzungen wurden sowohl inhaltsbasierte Codes im Sinne der Grounded Theory (Glaser und Strauss 1967) entwickelt als auch theoretische Codes hinzugefügt. Theoretische Codes wurden eingeführt, da die zeitlichen und personellen Ressourcen des Projekts nicht ausreichten, eine hinreichend fundierte Ableitung allein aus dem Material zu erreichen. Auf diese Weise wurde jedes Interview einmal codiert, wobei die Zitate (in MAXQDA: Segment) in der Länge variieren konnten. Die Auswertung richtete sich auf soziale Beziehungen (Beitrag Knabe et al., in diesem Heft), weshalb ein Zitat immer dann eröffnet wurde, sobald von einer dyadischen Beziehung gesprochen wurde. Wurde von verschiedenen Dyaden innerhalb von Absätzen gesprochen, konnten die Zitate überlappen. Entlang dieser Struktur wurden die Zitate im ersten Schritt in vivo codiert, d. h., die Urteiler*innen vergaben Codes, die ihr individuelles Verständnis des Zitats ausdrückten. In den Teamsitzungen wurden diese In-vivo-Codes schrittweise zu Kategorien zusammengefasst und hierarchisiert. Nach Fertigstellung der Codeliste Mitte Oktober 2020 wurden alle Interviews einmal „durchcodiert“, d. h., jedes Zitat wurde mit allen Codes versehen, die nach Meinung der Rater*innen zutrafen. Auf diese Weise sollte es möglich sein, das gemeinsame Auftreten von Codes zu analysieren und grobe Quantifizierungen vorzunehmen.

Über alle 19 Dokumente wurden 2942 In-vivo-Codes vergeben, von denen 500 auf die Gruppe der Haushalte ohne psychische Belastung (*n* = 4) und 2041 auf die Familien, in denen wenigstens ein psychisch erkranktes Kind wohnt (*n* = 12), entfallen. Die 3 Familien, in denen mindestens ein Elternteil krank ist, wurden mit 401 In-vivo-Codes versehen. Durch die Pandemie bedingte Probleme wurden 427-mal codiert, wobei 18 Kategorien gebildet wurden. Bewältigungshandeln wurde 378-mal codiert und in 15 verschiedene Strategien eingeteilt. Außerdem berichteten die Familien über unterschiedliche Ressourcen, die ihnen zur Verfügung standen (7 Kategorien mit 195 Codes) und darüber, wie sie die Entwicklung bis zum Zeitpunkt des Interviews beurteilten (*n* = 268 Codes in 4 Kategorien, beides nicht hier analysiert). Daneben wurden Kontrollstrategien erhoben (92 Codes, von denen 54 in der Kategorie „sekundäre Kontrolle“ und 17 der Kategorie „primäre Kontrolle“ zugeordnet werden konnten).

Für alle Kategorien stellten die Codierer*innen wöchentlich ihre In-vivo-Codierungen vor, die dann diskursiv kategorisiert wurden. Um die Arbeit abzukürzen, wurden für die Coping-Strategien heuristische Kategorien eingeführt, namentlich die 14 Skalen zum Bewältigungsverhalten aus dem Stressverarbeitungsfragebogen (SVF; Erdmann und Janke 2008), die um die Kategorie „Mediengebrauch“ ergänzt wurden.

## Ergebnisse

Um einen Überblick zu gewinnen, werden die einzelnen Problemklassen vorgestellt und dann in ihrer Nennhäufigkeit für die 3 Gruppen aufgelistet. Ähnlich wird mit den genannten Bewältigungsstrategien verfahren. Da Quantifizierungen in qualitativen Studien insbesondere von der Redseligkeit der Befragten abhängen, ist es angeraten, diesen Effekt zu kontrollieren, was über die Bildung von Quotienten modelliert wurde (Reis 2018).

### Problemlagen

Eine nach Häufigkeit geordnete Übersicht über die Problemlagen, die aus den 427 In-vivo-Codes verdichtet wurden, gibt Tab. 1. Erwartungsgemäß zeigt sich, dass es keine Familie gibt, die nicht über Probleme berichtet. Die Quotienten, gebildet aus der Zahl der Problemcodes und der Zahl der Familien, rangieren zwischen 18,5 und 23,9 (Tab. 1, *erste Zeile*), d. h., die 3 Familientypen sind hinsichtlich der Berichtsmasse vergleichsweise homogen, wobei die Familien mit kranken Mitgliedern etwas mehr berichten.

**Table 1:** 

Probleme	Anzahl der In-vivo-Codes (*n* = 427)	Familie ohne Störung 74 (4) ≙ *18,5*	Familie mit erkranktem Kind 287 (12) ≙ *23,9*	Familie mit erkranktem Elternteil 65 (3) ≙ *21,6*
Corona unspezifisch	108	26 (4) ≙ *6,5*	71 (12) ≙ *5,9*	11 (2) ≙ *5,5*
Coronaeinschränkungen	104	15 (4) ≙ *3,75*	78 (12) ≙ *6,5*	11 (3) ≙ *3,7*
Stress	65	11 (4) ≙ *2,75*	44 (10) ≙ *4,4*	10 (3) ≙ *3,3*
Homeschooling	53	3 (1) ≙ *3*	43 (11) ≙ *3,9*	7 (2) ≙ 3,5
Soziale Verluste	43	13 (4) ≙ *3,2*	22 (9) ≙ *2,4*	8 (2) ≙ *4*
Mehrfachbelastung	39	7 (2) ≙ *3,5*	29 (8) ≙ *3,6*	3 (2) ≙ *1,5*
Fehlende Struktur	30	12 (3) ≙ *4*	12 (8) ≙ *1,5*	6 (1) ≙ *6*
Fehlende soziale Unterstützung	29	4 (2) ≙ *2*	13 (7) ≙ *1,8*	12 (3) ≙ *4*
Gesundheit	23	4 (2) ≙ *2*	11 (5) ≙ *2,2*	8 (1) ≙ *8*
Enge	19	4 (2) ≙ *2*	14 (6) ≙ *2,3*	1 (1) ≙ *1*
Existenzielle Bedrohung	11	2 (1) ≙ *2*	7 (4) ≙ *1,75*	2 (1) ≙ *2*
Sozialer Druck	8	0	8 (3) ≙ *2,7*	0
Finanzielle Probleme	6	1 (1) ≙ *1*	4 (2) ≙ *2*	1 (1) ≙ *1*

*In Klammern* Anzahl (*n*) der Familien mit Nennungen, *kursiv* Quotient aus der Zahl der Codes und der Zahl der Familien

Dieses Muster betrifft auch jene 108 unspezifischen Probleme, die zwar mit der Pandemie zusammenhängen, aber bis zum Ende des Projekts nicht anders zugeordnet wurden. Hierzu gehören sehr unterschiedliche Probleme, die von einem verunmöglichten Büfett im Garten zur Jugendweihe des Sohnes (E01§121) über „negatives Denken“ (K04§380) bis zu schwierigen Umzügen (K06§428) reichen. Unerwartete Probleme entstanden, wenn etwa durch die Belastungssituationen Familiengeheimnisse, wie außerehelichen Beziehungen, auftauchten: „Dadurch, dass ich auch im Krankenhaus war und er sich richtig Sorgen gemacht hat, gab’s auch nochmal ’ne andere Situation, [in der] bei ihm noch Sachen rauskamen, die vorher noch nicht so ganz klar waren“ (K07$310). Die unspezifischen Belastungen jedoch verteilten sich ungefähr gleich über die untersuchten Gruppen und ließen keine Sonderstellung der Familien mit erkrankten Mitgliedern erkennen. Probleme, die unter „Coronaeinschränkungen“ zusammengefasst wurden, betreffen hingegen die Familien mit erkrankten Kindern deutlicher als die anderen Typen, fast doppelt so häufig (6,5:3,7). Eine nähere Inspektion ergibt, dass beispielsweise Aggressionen zwischen den Kindern in dieser Gruppe etwas häufiger berichtet werden als in den anderen beiden. Eine Mutter führt dies auf den Mangel an außerhäuslichen Kontakten der Kinder zurück: „Die Zeit, wo es hieß, die Kiddis dürfen keine Freunde treffen oder so, dann waren halt die Großen immer aufeinander, und der Große braucht halt auch ab und zu mal Zeit für sich, und klar, der ist jetzt auch zwölf, und das hat die Achtjährige immer nicht verstanden, die ist immer wieder rein und hat genervt und dann gab’s irgendwann auch mal ’ne kleine Klopperei, und das hat man dann schon fast jeden Tag …“ (K01§57). Auch aus diesem Grund stellte sich das Allein-zu-Haus-Lassen der Kinder in vielen Familien mit erkrankten Kindern zumindest anfänglich als größtes Problem heraus, insbesondere, wenn die Eltern ihrer Arbeit nachgehen mussten: „… dass sie keinen Blödsinn macht, auf Deutsch gesagt … Es ist natürlich beruhigend, wenn man weiß, Kind geht morgens zur Schule, und es kommt zu seinen normalen Zeiten wieder nach Hause“ (K04§119). Auch der Ausfall sportlicher Betätigung im Verein traf die Familien mit erkrankten Kindern härter als die anderen, trotz elterlicher Anstrengungen: „Am Anfang … bin ich mit ihr zusammen gegangen, irgendwann hatte sie keine Lust mehr, also Lauftraining machen, Kickboxen, ne. Das ist alles ausgefallen. auch noch Kampfsport, ne, das ist alles ausgefallen, und das … fand ich sehr schade“ (K06§100). Auffällig sind auch Nennungen, in denen Eltern mit erkrankten Kindern die Probleme beschreiben, die entstanden, wenn sie ihre Kinder zu wichtigen Verrichtungen mitnehmen müssen, und diese größere Probleme mit der Maskenpflicht hatten, etwa beim Einkaufen.

Auch bei den Codes, die unter „Stress“ zusammengefasst wurden, fallen die Eltern psychisch erkrankter Kinder mit häufigeren Nennungen auf. Die meisten Stresscodes waren an Einschränkungen gebunden, u. a., weil den Eltern psychisch erkrankter Kinder die Zeit zum Umlernen zu kurz war. Die Probleme, die sich v. a. auf das Homeschooling beziehen (*n* = 53), verteilen sich hingegen gleichmäßig auf die Gruppen. Der letzte deutliche Unterschied, der erörtert werden soll, betrifft die „fehlende soziale Unterstützung“. Alle erkrankten Eltern beklagen sich massiv, während die anderen Familien hierzu seltener berichten. Die dahinterstehenden Gründe sollen kurz inspiziert werden. Einweisungen in die Klinik, die aus der Sicht der Familien mit der Pandemie zusammenhängen, erschwerten das Familienleben sehr. In diesem Fall berichtet die Mutter: „… es war eher so dieses, ja so Ärger und Wut, und dass alles so extrem schwierig dadurch wird, also so ich hab mir gedacht, jetzt die Zeit ist sowieso schon schwierig für die Kinder und für meinen Mann, wenn ich jetzt in der Klinik bin …“ (E01§111). Auch eine andere Patientin, die zusätzlich somatisch erkrankt ist, darf eigentlich nicht krank werden, da ohne funktionierende Einrichtungen die Betreuung ihres jüngsten Kindes nicht gewährleistet ist: „Ne, denn auch noch hier mit’m Krankenhaus … mit Herzkatheter, war auch die Coronazeit. So, wo bleibt er jetzt, mein [kleiner] Sohn … mein großer Sohn konnte ihn nicht nehmen …“ (E02§70). Als alleinerziehende Mutter ohne Freundesnetzwerk und mit nur wenigen, kaum belastbaren Beziehungen hat die Befragte einen starken Konflikt: „Freunde hab’ ich keine, ich sach mal, ich hab nur ’ne gute Bekannte … oder ein älteren Herrn, aber er wurde jetzt auch schwer krank … er raucht zu viel, und ich kann das nicht ab. Und meine Bekannte, mit der ich Kaffee trinken war, der kannste nicht alles erzählen … das sind dann keine Freunde“ (E02§86). Einer dritten Patientin, die von ihrem Mann getrennt lebte, wurden die Coronaregelungen zum Verhängnis, die sich eher auf Haushalte, aber nicht auf Familien, richteten. Ihr geschiedener Mann argumentierte für den vollständigen Verbleib der Kinder bei ihm, da sie, als arbeitende Mutter mit weniger Platz in Pandemiezeiten nicht ausreichend für die Gesundheit der Kinder sorgen könne. Alle 3 Patientinnen fühlten sich mit derartigen Problemen alleingelassen, da diese auch nicht „wegtherapiert“ werden könnten. Hiermit beschrieben sie den Umstand, dass sie die COVID-19-bezogenen Belastungen als unabhängig von ihrer Krankheit und damit der Psychotherapie unzugänglich erlebten.

Zusammenfassend lassen sich zwei Komplexe ausmachen, die familientypisch zu sein scheinen. Familien mit erkrankten Kindern leiden deutlicher unter den Einschränkungen, die sich jedoch weniger auf das Homeschooling beziehen, sondern eher auf die fehlenden Möglichkeiten außerhalb der Familie. Die fehlende Betreuung machte es allen Eltern schwer, täglich zur Arbeit zu gehen. Bei psychisch erkrankten Eltern stehen fehlende Unterstützungen der Eltern selbst im Vordergrund.

### Bewältigungsstrategien

Die Nennungen zu Bewältigungsstrategien wurden ähnlich quantifiziert und geordnet wie die zu Problemen. Der Übersicht halber werden in Tab. 2 nur Strategien aufgeführt, die mindestens 7‑mal genannt wurden. Grundsätzlich lässt sich auch hier sagen, dass die pandemiebedingten Probleme (Tab. 1, *erste Zeile*) auf vielfältigste Weise bewältigt werden, wobei wieder die Familien mit erkrankten Mitgliedern etwas mehr berichten.

**Table 2:** 

Bewältigungsstrategien	Anzahl der In-vivo-Codes (*n* = 378)	Familie ohne Störung 65 (4) ≙ *16,25*	Familie mit erkranktem Kind 258 (12) ≙ *21,5*	Familie mit erkranktem Elternteil 55 (3) ≙ *18,3*
Situationskontrolle	99	11 (2) ≙ *5,5*	72 (11) ≙ *6,5*	11 (2) ≙ *5,5*
Suche nach sozialer Unterstützung	76	17 (2) ≙ *8,5*	45 (11) ≙ *4,1*	14 (3) ≙ *4,7*
Gedankliche Weiterbeschäftigung	41	3 (2) ≙ *1,3*	26 (6) ≙ *4,3*	12 (2) ≙ *6*
Ersatzbefriedigung	42	5 (2) ≙ *2,5*	26 (8) ≙ *3,2*	11 (3) ≙ *3,7*
Vermeidung	30	8 (4) ≙ *2*	22 (9) ≙ *2,4*	0
Positive Selbstinstruktion	26	9 (3) ≙ *3*	15 (8) ≙ *1,9*	2 (2) ≙ *1*
Herunterspielen durch Vergleich mit anderen	24	9 (4) ≙ *2,2*	14 (8) ≙ *1,8*	1 (1) ≙ *1*
Schuldabwehr	18	0	14 (3) ≙ *4,7*	4 (1) ≙ *4*
Bagatellisierung	16	3 (2) ≙ *1,5*	12 (8) ≙ *1,5*	1 (1) ≙ *1*
Selbstbemitleidung	15	0	13 (5) ≙ *2,6*	2 (1) ≙ *2*
Aggression	15	1 (1) ≙ *1*	13 (5) ≙ *2,6*	1 (1) ≙ *1*
Flucht	12	2 (1) ≙ *2*	8 (7) ≙ *1,1*	2 (1) ≙ *2*
Resignation	12	1 (1) ≙ *1*	11 (6) ≙ *1,8*	0
Entspannung	12	3 (2) ≙ *1,3*	5 (4) ≙ *1,2*	4 (1) ≙ *4*
Mediennutzung	9	2 (1) ≙ *2*	7 (3) ≙ *2,3*	0
Ablenkung	7	0	6 (4) ≙ *1,5*	1 (1) ≙ *1*
Soziale Abkapselung	7	1 (1) ≙ *1*	4 (2) ≙ *2*	2 (1) ≙ *2*

*In Klammern* Anzahl der Familien mit Nennungen, *kursiv* Quotient aus Zahl der Codes und Zahl der Familien

Die meisten Nennungen ließen sich als Ansätze kategorisieren, die Pandemiesituation unter Kontrolle zu bekommen, soweit dies möglich war. Dazu gehörte die fortwährende Motivation der Kinder: „Da fing er mit mal an. Ne, ich hab keine Lust, Hausaufgaben zu machen! Ich sach, das muss doch gemacht werden, sach ich, auch wenn’s mir nicht gutgeht, müssen wir doch trotzdem die Hausaufgaben machen, ne. Dann ham wir Stück für Stück dann mal zwei Blätter, am nächsten Tag dann mal drei Blätter … Ich hab ihn dann immer motiviert …“ (E02§70). In Tab. 2 wird gezeigt, dass sich hier die Ratio der Familientypen kaum unterscheidet, weshalb auf diese aktive Art der Bewältigung nicht näher eingegangen wird. Für die zweithäufigste Art der Bewältigung, die Suche nach sozialer Unterstützung, unterscheiden sich die Gruppen erheblich. Zwar berichtet nur die Hälfte der gesunden Familien hierzu pandemiebezogene Bewältigungsstrategien in unterschiedlichen Familientypen, dann jedoch doppelt so häufig wie die Familien mit erkrankten Mitgliedern. Ein entgegengesetztes Bild bietet sich für Ruminationen, im SVF „gedankliche Weiterbeschäftigung“ genannt. Hier sind die Berichte aus den kranken Familien massiver, wobei die kranken Eltern besondere Betroffenheit signalisieren. Hier betrafen die Ruminationen das Zusammentreffen von eigener Erkrankung, Pandemie und Elternrolle. Mitunter äußerte sich die Sorge schon in den ersten Momenten: „wie mein Sohn dann sachte, ja, wir gehn ab Freitag nicht mehr zur Schule. Oh Gott, hab ich gesagt, na, was das wohl wird, … wir werden dann wahrscheinlich immer aneinandergeraten“ (E02§66).

Diese Ängste wurden noch gesteigert, wenn die Hilfemöglichkeiten durch die Pandemie ebenso verringert wurden: „… und wenn du deine Panik- und Angstattacken dann hattest, wo gehste jetzt hin? Ins Krankenhaus traute ich mir schon gar nicht“ (E02§80). In den Familien mit erkrankten Kindern kreisten die Gedanken häufiger um die Kinder. So blieb die Angst vor Ansteckungen lange präsent. Mehrere Familien mit erkrankten Kindern berichteten, dass sie sich ständig das Ende der Beschränkungen herbeiwünschten: „Also, man ersehnt sich doch wirklich den ganz normalen Wahnsinn wieder, ne, also nicht diesen, ja, wie soll man das ausdrücken, ne, diesen … Es ist eigentlich alles mit Stress verbunden“ (K04§89). Oft liegen die lastenden Gedanken bei den Kindern selbst: „Sie macht sich unwahrscheinlich, sehr viel Sorgen: ‚Könn’ wir krank werden, könn’ wir nicht krank werden?‘. Hauptproblem ist, dass sie sich eigentlich den ganzen Tag damit beschäftigt, wie’s uns geht“ (K04§160).

Eine andere Mutter eines erkrankten Kindes wird die Gedanken nicht los, dass die Auswirkungen des Lockdowns für ihren Sohn anhaltend sein werden: „Ja, und er hat halt viele Sachen auch wieder verlernt, die er vorher schon konnte, wo jetzt auch wieder mehr Arbeit ist, um das wieder zu lernen, das ist ganz schwierig, so gerade in der Schule, sich wieder daran zu erinnern, beim Schreiben nicht mitzusprechen, beim Unterricht zuzuhören, nicht selber zu sprechen, ne, und dann ist es hier natürlich noch schwieriger, ihn so zu händeln, und das wird uns nächstes Jahr genauso kommen … Das ist nicht gut, definitiv nicht“ (K06§226).

Von allen anderen Bewältigungsstrategien ist der Unterschied bei „Schuldabwehr“ noch offensichtlich, wobei alle Nennungen in den kranken Familien liegen. Die meisten dieser Codes beschreiben die Externalisierung von Schuld für Probleme, wofür im Fall der Familien mit erkrankten Kindern häufig diese selbst herhalten mussten. In den Familien mit erwachsenen Patientinnen wurden häufiger andere Erwachsene oder Institutionen als Schuldige benannt.

## Diskussion

Diese Studie verfolgte das Ziel, mithilfe eines „entdeckenden“ qualitativen Ansatzes besondere Problemlagen und Bewältigungsstrategien in Familien mit psychisch erkrankten Mitgliedern , die während des ersten Lockdowns auftraten, zu identifizieren. Mithilfe einer quantifizierenden Analyse sollten Problemlagen und Bewältigungsformen identifiziert werden, die Familien mit psychisch erkrankten Mitgliedern besonders betreffen könnten. Auf die schnelle Verbreitung des Virus und die zeitnahe Einführung der Maßnahmen musste schnell und kostengünstig reagiert werden, weshalb in hoher Geschwindigkeit eine Studie aufgelegt wurde, die damit einige Limitationen in Kauf nahm. Trotz der methodischen Einschränkungen können im Folgenden einige Resultate festgehalten und interpretiert werden.

Die Maßnahmen zur Bekämpfung der Pandemie während des ersten Lockdowns bedeuteten erhebliche Unterbrechungen für *alle* Eltern, unabhängig davon, ob sie oder ihre Kinder zusätzliche psychische Probleme hatten oder nicht. Dieses Ergebnis liegt in der Natur der Maßnahmen – sie schränkten den Alltag grundsätzlich und für alle ein, und „objektive“ Faktoren wie Wohnungsgröße, Familienstruktur und Einkommen wirken vermutlich stärker auf Probleme und Bewältigung als das Vorliegen einer psychischen Erkrankung. Eine wichtige Einschränkung der Studie besteht darin, dass die vorgelegten Analysen soziale Risiken wie Armut, prekäre Beschäftigung, hohe Wohndichte und viele andere ausklammern mussten. Wie aus diversen Studien bekannt ist, sind soziale Risiken und psychische Erkrankung nicht unabhängig voneinander. Für die Darstellung eines Falls, in dem auch eine wohlhabende Familie an die Grenzen ihrer Belastbarkeit kam, wird auf die Studie von Knabe et al. (in diesem Heft) am gleichen Datensatz verweisen. Doch die Aufklärung des Zusammenspiels von sozialen Risiken und psychischer Erkrankung war nicht vordringliches Ziel der Untersuchung. Vielmehr ging es um die Identifikation prominenter Problemlagen für Familien mit psychisch erkrankten Mitgliedern, die im Design zukünftiger Studien enthalten sein sollten.

Danach unterscheiden sich Familien mit erkrankten Mitgliedern nur an einigen, aber wichtigen Stellen von Familien ohne erkrankte Mitglieder. Die Einschränkungen treffen die psychisch erkrankten Kinder demnach stärker im Bereich der außerschulischen Kontakte, die vorher als Ventil dienen konnten – Sportvereine und Freundschaften. Das größte Risiko am Homeschooling ist für die erkrankten Kinder der mögliche Verlust an extern vorgegebener Tagesstruktur und Regel. Probleme der Beschulung selbst sind eher abhängig von der verfügbaren Elternzeit, dem Platz und den kognitiven Ressourcen in der Familie. Problematisch ist grundsätzlich der Wegfall der Betreuung, wenn Eltern dadurch ihrer Arbeit nicht nachgehen können. Salopp gesagt: Der drohende Verlust des Arbeitsplatzes wog hier schwerer als ein wiederholtes Schuljahr. Hier spielen soziale und Krankheitsrisiken unmittelbar zusammen, wobei ein wichtiger mediierender oder moderierender Mechanismus die (dysfunktionalen) Geschwisterbeziehungen zu sein scheinen.

Der Wegfall sozialer Unterstützung betrifft psychisch erkrankte Eltern insbesondere dann, wenn deren Probleme sich als nahezu unlösbar erweisen und Behandlungsbedürftigkeit ihrer Erkrankung und Schutzbedürftigkeit ihrer Kinder aufeinanderprallen. Eltern mit psychischen Erkrankungen verfügten über weniger soziale Unterstützung, weshalb sie sich als extrem abhängig von institutioneller Unterstützung erwiesen. Hier besteht also ein Doppelbedarf – für die Eltern und die Kinder, der durch die Pandemie erheblich vergrößert wurde.

Was die Bewältigungsstrategien angeht, fallen die Familien mit psychischen Erkrankungen insbesondere durch Strategien auf, die sich eher auf Emotionen statt auf Situationen richten und häufig negative Valenzen haben. Insbesondere Ruminationen treten massiv auf, ebenso wie Abwehrhaltungen. Mehrfach wurde darauf hingewiesen, dass mit der Krise diese Sorgen gewissermaßen „objektiviert“ würden und sich damit der Psychotherapie entzögen.

Was die Versuche angeht, der angespannten Situationen Herr zu werden, sind sich die Familien eher ähnlich. Unabhängig davon, ob die Familie erkrankte Mitglieder hat oder nicht, streben die Familien danach, die Situationen zu kontrollieren. In allen Familien gaben die Eltern an, unter der Aussicht einer Wiederholung und/oder Verlängerung des Lockdowns zu leiden, da Homeschooling auf Dauer kaum mit einer Erwerbstätigkeit zu vereinbaren sei, egal ob diese zu Hause oder in der Firma ausgeübt würde. Unterschiede traten eher bei emotionszentrierten Strategien auf, die von Familien mit kranken Mitgliedern genutzt werden. Schuldzuweisungen innerhalb der Familie betrafen bei Familien mit erkrankten Kindern häufig die Kinder, bei erkrankten Erwachsenen häufiger die Institutionen, einschließlich der Therapeut*innen.

Abschließend sollen weitere Einschränkungen genannt werden, die den Aussagewert der Studie begrenzen, – die allesamt den besonderen Umständen der Studie geschuldet waren. So wurden die Interviews von klinisch unerfahrenen Soziologiestudent*innen durchgeführt, die trotz intensiver Betreuung mitunter vor der „ungeschminkten Wirklichkeit“ an Distanz einbüßten, wenn es beispielsweise um Schilderungen häuslicher Gewalt ging. Auch die Patient*innenfamilien ließen sich trotz der Incentives schwerer zur Mitarbeit bewegen als anfänglich gedacht, womit eine nur unausgewogene Verteilung über die Familientypen erreicht werden konnte. Schließlich musste die Studie mit dem Auslaufen des Semesters abgebrochen werden, da danach nur vereinzelt Interviewer*innen zur Verfügung standen. Doch auch eingedenk dieser Limitationen konnte ein Datensatz hergestellt werden, der in mehrfacher Hinsicht auswertbar ist (Knabe et al., in diesem Heft) und wichtige Hinweise für die Formulierung weiterführender Forschungsfragen enthält. Danach stellen Familien mit psychisch erkrankten Mitgliedern eine vulnerable Gruppe dar, in der die vorhandenen Risiken durch die Pandemie in ihrer Wirkung verstärkt werden. Familiäre Problemlagen und Regulationen sind davon nicht ausgenommen. Mit diesem Mechanismus der *Akzentuierung* wirkt die gegenwärtige Pandemie auf Familien vermutlich ähnlich wie die deutsche Vereinigung auf ostdeutsche Familien (Reis 2018). Idealerweise sollten daher Familien mit psychisch erkrankten Mitgliedern in prospektiven Längsschnittstudien erfasst werden, zumal nicht absehbar ist, wann und wie ähnliche Krisen ähnliche Regulationen von ihnen erfordern.

## Fazit für die Praxis

- Obwohl Familien mit psychischen Störungen der Pandemie insgesamt einsamer gegenüberstehen, mobilisieren sie weniger soziale Unterstützung.
- Ruminationen sind wichtigster Teil eines eher emotionszentrierten Coping.
- Für psychisch kranke Kinder wird das Problem der Beschulung vom Wegfall der Tagesstruktur sowie außerfamiliärer Kontakte und Aktivitäten noch übertroffen.
- Für Familien mit psychischen Problemen sollten Extraangebote zum „healthy parenting“ unter Stress gemacht werden.
